# *Pistacia chinensis* Methanolic Extract Attenuated MAPK and Akt Phosphorylations in ADP Stimulated Rat Platelets *In Vitro*

**DOI:** 10.1155/2012/895729

**Published:** 2012-07-30

**Authors:** Ji Young Park, Mei Hong, Qi Jia, Young-Chul Lee, Taddesse Yayeh, Eujin Hyun, Dong-Mi Kwak, Jae Youl Cho, Man Hee Rhee

**Affiliations:** ^1^Department of Veterinary Medicine, College of Veterinary Medicine and Stem Cell Research Therapeutic Institute, Kyungpook National University, Daegu 702-701, Republic of Korea; ^2^Unigen Inc., Chungnam, Cheonan 330-863, Republic of Korea; ^3^Department of Genetic Engineering, Sungkyunkwan University, Suwon 440-746, Republic of Korea

## Abstract

*Pistacia chinensis* (Chinese pistache) is a widely grown plant in southern China where the galls extract is a common practice in folk medicine. However, extracts from this plant have never been attempted for their cardiovascular protective effects in experimental setting. Here therefore we aimed to investigate the antiplatelet activity of *Pistacia chinensis* methanolic extract (PCME) in ADP stimulated rat platelets *in vitro*. PCME (2.5–20 *μ*g/mL) inhibited ADP-induced platelet aggregation. While PCME diminished [Ca^2+^]*i*, ATP, and TXA2 release in ADP-activated platelets, it enhanced cAMP production in resting platelets. Likewise, PCME inhibited fibrinogen binding to **α**IIb**β**3 and downregulated JNK, ERK, and Akt phosphorylations. Thus, PCME contains potential antiplatelet compounds that could be deployed for their therapeutic values in cardiovascular pathology.

## 1. Introduction

Platelets adhere and mitigate bleeding during vascular injury through plug formation [1]; nevertheless, their dysfunctions gear toward thrombus development and subsequent cardiovascular pathology [2]. At the injured vascular site, the exposed subendothelial collagen and von Willebrand factor (vWF) initiate platelet activation marked by the secretion of prothrombotic factors like ADP, thrombin, serotonin, and thromboxane A2 (TXA2) [3]. Platelet activation is also further amplified by dense granule secretions (ADP and ATP) and *α*-granule adhesive molecules like fibrinogen and *P*-selectin [4].

ADP activates G-protein-coupled receptor (GPCR) signaling mediated by two metabotropic purinergic receptors in platelets, Gq-coupled P2Y_1_ receptor activates phospholipase C*β* whereas Gi-coupled P2Y_12_ receptor inhibits adenylyl cyclase and activates PI-3K/Akt and ERK [5]. Adenylyl cyclase enhances cyclic adenosine monophosphoate (cAMP) which activates protein kinase (PKA) that inhibits platelet shape change, granule secretion, calcium mobilization, and aggregation [6]. However, ADP-induced adenylyl cyclase attenuation vanishes cAMP production which culminate in platelet activation. Likewise, ADP-stimulated Gi-coupled P2Y12 receptor that activates mitogen-activated protein kinases (MAPKs) (ERK, JNK, and p38) and PI-3k/Akt signaling to arrive at similar scenario of platelet activation and aggregation [7]. 

*Pistacia chinensis* (chinese pistache) is a drought resistance deciduous indigenous tree in southern China. The tree is marked by its finely divided dark green foliage with an attractive orange bark which has been used frequently for grafting the commercial *Pistacia vera* [8] that showed antiatherosclerosis [9], anti-inflammatory [10], and antioxidant effects [11]. Nevertheless, the antiplatelet activity of *Pistacia chinensis* was unattempted yet. We therefore reported that *Pistacia chinensis* methanolic extract (PCME) inhibited ADP stimulated rat platelet granule secretions and aggregation *in vitro*. Furthermore, PCME attenuated ERK, JNK, and Akt phosphorylations in ADP-stimulated platelets, while it enhanced cAMP production.

## 2. Materials and Methods

### 2.1. Materials

Fura-2/AM was obtained from Sigma (St. Louis, MO, USA). ADP was procured from Chrono-log (Havertown, PA, USA). Antibodies against phospho-p44/42, p44/42, phospho-p38, p38, phospho-SAPK/JNK, and *β*-actin were bought from Cell Signaling (Beverly, MA, USA). ATP assay kits were purchased from Biomedical Research Service Center (Buffalo, NY, USA). TXB2 EIA was purchased from Enzo Life Sciences (Plymouth Meeting, PA, USA). cAMP enzyme immunoassay (EIA) kits were from Cayman Chemical (MI, USA). Fibrinogen Alexa Fluor 488 conjugate was obtained from Invitrogen molecular probes (Eugene, OR, USA). All other chemicals were of reagent grade.

### 2.2. Sample Preparation and GC Mass Spectrometry Analysis

**Figure 1:**
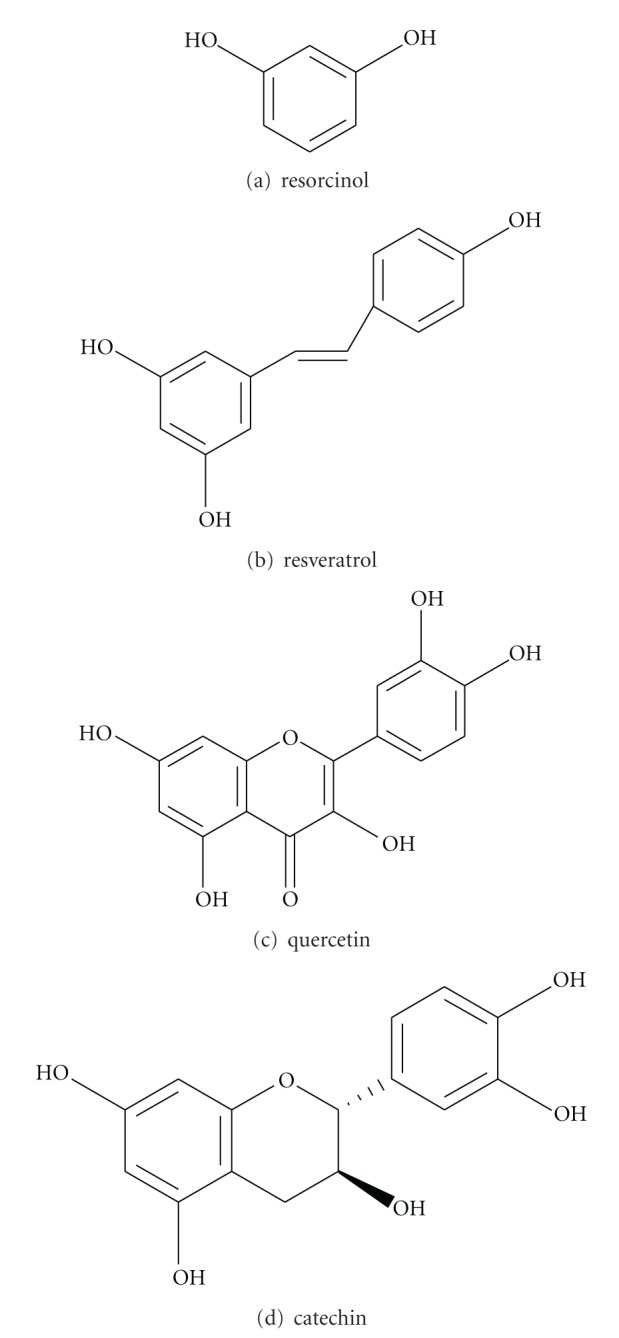
Structural analysis of major chemical compounds available in PCME.

Dr. Zhengliu Zhang identified* Pistacia chinensis* trunk bark collected from Sichuan, China and the voucher specimen (PLSA1301) was deposited at the Unigen Inc., till the extraction date. Trunk bark of *Pistacia chinensis* was dried and pulverized into powder, of which 159 g with 50% methanol/dichloromethane (MeOH/DCM) extraction yielded 11 g for silica gel fractionation to get 590 mg of subfraction. 300 mg of 500 mg was again further purified by preparative HPLC (Luna C18, 30 × 250 mm) to obtain 30 mg of fraction. PCME of the final fraction (2.5 mg) was employed in GC mass analysis indicating its polyphenolic nature replete with resorcinol (18.9%) and pyridine (2.8%) compounds with common structural moieties (Table 1 and Figure 1) except solvents like methane, DMSO, and dichloroethylene. GC mass spectrometry was performed using Agilent Technology 7890A-Gas Chromatograph system (Agilent Technologies, Santa Clara, CA, USA), coupled to XLMSD-5975C equipment operating in electrospray ionisation (EI) mode. 

### 2.3. Platelet Preparation and Aggregation Assay

Platelets preparations from rats were described previously [12]. Briefly, 8 weeks old male Sprague Dawely rats (240–250 g weight) were obtained from Orient Co. (Seoul, Korea) and maintained in a standard laboratory facility with *ad libitum* feed and water. Whole blood was collected using a 23G needle from abdominal aorta and then transferred into 15 mL test tube containing 1 mL of the anticoagulant acid/citrate/dextrose (ACD, 85 mM trisodium citrate, 83 mM dextrose, and 21 mM citric acid). Blood was centrifuged at 170 ×g for 7 min to obtain platelet-rich plasma which was further centrifuged at 120 ×g for 7 min to remove residual erythrocytes. This platelet-rich plasma was centrifuged twice at 350 ×g with a washing buffer for 10 min to remove the ACD solution, and then platelet precipitates were adjusted to (3 × 10^8^/mL) for aggregation assay in Tyrode buffer (137 mM of NaCl, 12 mM of NaHCO_3_, 5.5 mM of glucose, 2 mM of KCl, 1 mM of MgCl_2_, 0.3 mM of NaHPO_4_, and pH 7.4). All platelet preparations were conducted at room temperature, and all experimental procedures and protocols used in this investigation were reviewed and approved by the Ethics Committee of the College of Veterinary Medicine, Kyungpook National University.

Platelet aggregation was conducted as mentioned before. Briefly, aggregation was monitored by measuring light transmission in an aggregometer (Chronolog, Havertown, PA, USA). The washed platelets were preincubated at 37°C for 2 min with either PCME or vehicle (<0.1%), and then stimulated with agonists. The reaction mixture was further incubated for 5 min, stirring at 170 ×g. 

### 2.4. Determining [Ca^2+^]*i*

The [Ca^2+^]*i* was determined with Fura-2/AM as described previously [13]. Briefly, platelets were incubated with 5 *μ*M of Fura-2/AM for 30 min at 37°C and washed. The Fura-2-loaded platelets (3 × 10^8^/ml) were then pre-incubated with PCME for 3 min at 37°C in the presence of 1 mM CaCl_2_, then stimulated with ADP for 5 min. Fluorescence signals were recorded using a Hitachi F-2500 fluorescence spectrofluorometer (F-2500, Hitachi, Japan). Fluorescence emission was determined at 510 nm, with simultaneous excitation at 340 and 380 nm, changing every 0.5 s. Fura-2 fluorescence was measured in a spectrofluorometer by the method of Schaeffer: [Ca^2+^]*i* in cytosol = 224 nM × (*F* − *F*_min⁡_)/(*F*_max⁡_ − *F*), where 224 nM is the dissociation constant of the Fura-2-Ca^2+^ complex, and *F*_min⁡_ and *F*_max⁡_ represent the fluorescence intensity levels at very low and very high Ca^2+^ concentrations, respectively. In our experiment, *F*_max⁡_ is the fluorescence intensity of the Fura-2-Ca^2+^ complex at 510 nm after the platelet suspension containing 1 mM of CaCl_2_ had been solubilized by Triton X-100 (0.1%). *F*_min⁡_ is the fluorescence intensity of the Fura-2-Ca^2+^ complex at 510 nm, after the platelet suspension containing 20 mM Tris/3 mM of EGTA had been solubilized by Triton X-100 (0.1%). *F* represents the fluorescence intensity of the Fura-2-complex at 510 nm after the platelet suspension was stimulated by ADP, with and without PCME, in the presence of 1 mM CaCl_2_. 

### 2.5. ATP Release Assay

Washed platelets were pre-incubated for 2 min at 37°C with various concentrations of PCME and then platelets were stimulated with ADP for 5 min. After the reaction was terminated, samples were centrifuged and supernatants were used for ATP assay in a luminometer (GloMax 20/20, Promega, Madison, USA) using an ATP assay kit (Biomedical Research Service Center, Buffalo, USA).

### 2.6. Determination of Thromboxane A2 Generation

Washed platelets (3 × 10^8^/ml) were pre-incubated with or without PCME for 2 min in the presence of 1 mM CaCl_2_, and then the platelets were stimulated with ADP. Reactions were terminated after 5 min by adding ice-cold 2.5 mM EDTA and 100 *μ*M indomethacin. After centrifugation at 14,000 rpm for 3 min at 4°C, the amount of TXB2 (supernatant) was determined using TXB2 EIA kit according to the manufacturer's protocol (Enzo Life Sciences, Plymouth Meeting, PA, USA).

### 2.7. Measurements of cAMP

Washed platelets were pre-incubated for 2 min at 37°C with various concentrations of PCME or vehicle in the presence of 1 mM CaCl_2_, and then stimulated with ADP for 5 min in a platelet aggregometer. The reaction was terminated by the addition of equal volumes of 80% ice-cold ethanol. Samples were then centrifuged at 2,000 ×g for 10 min at 4°C and the supernatant cAMP level was determined with a cyclic AMP (Ann Arbor, MI, USA).

### 2.8. Immunoblotting

Proteins were prepared as previously described [14]. Briefly, platelet suspensions (3 × 10^8^/mL) were pre-incubated with PCME or vehicle. Platelet activation was induced by ADP and the reaction was allowed to proceed for 5 min. After termination of the reaction, lysates were prepared by solubilizing and centrifuging platelets in sample buffer (0.125 M Tris-HCl at pH 6.8, 2% SDS, 2%  *β*-mercaptoethanol, 20% glycerol, and 0.02% bromophenol blue) in the presence of protease inhibitors (*μ*g/ml: 1 phenylmethylsulfonylfluoride (PMSF), 2 aprotinin, 1 leupeptin, and 1 pepstatin A). Protein concentration was determined using BCA Assay (PRO-MEASURE, iNtRON Biotechnology, Korea). 30 *μ*g of protein was separated in 10% SDS-PAGE and transferred to nitrocellulose membrane in transfer buffer (25 mM Tris (pH 8.5), 0.2 M glycine, and 20% methanol). Immunoblots were blocked with TBS-T containing 5% non-fat dry milk, washed, and incubated with primary antibody diluted in a blocking solution. The immunoblots were again probed with horseradish peroxidase secondary antibody, and membranes were visualized using enhanced chemiluminescence, ECL (iNtRON Biotechnology, Korea).

### 2.9. Fibrinogen-Binding Assay

Alexa Fluor 488-fibrinogen binding to the washed platelets was quantified by flow cytometry. Briefly, the washed platelets were pre-incubated with PCME at room temperature in the presence of 0.1 mM CaCl_2_. The platelets were then stimulated by ADP for 5 min, and immediately incubated with Alexa Fluor 488-human fibrinogen (20 *μ*g/mL) for 5 min and fixed with 0.5% paraformaldehyde at 4°C for 30 min. Platelets were pelleted by centrifugation at 2000 ×g at 4°C and resuspended in 500 *μ*L PBS. Binding of fibrinogen to integrin *α*_IIb_ *β*_3_ was measured in the presence of calcium chelator EGTA 1 mM. Fluorescent intensities were determined using FACS Calibur cytometer (BD Biosciences, San Jose, UAS), and data were analyzed using CellQuest software (Becton Dickinson Immunocytometry Systems, San Jose, CA).

### 2.10. Statistical Analysis

One-way analysis of variance was used for data analysis followed by a post hoc Dunnett's test for statistical significance. All data were means ± S.E.M. *P* values less than 0.05 were considered statistically significant.

## 3. Results

### 3.1. PCME Inhibited ADP-Induced Platelet Aggregation

**Figure 2:**
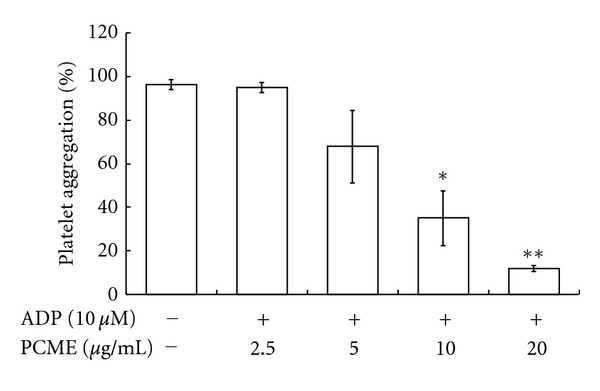
The inhibitory effect of PCME in ADP-induced platelet aggregation. Platelets (3 × 10^8^/ml) were preincubated with or without PCME in the presence of 1 mM CaCl_2_ for 3 min at 37°C and then stimulated with 10 *μ*M ADP. Agonist-induced platelet aggregation was terminated at 5 min, and then percent aggregation rate was determined. Each graph shows mean ± SEM from 4 independent experiments performed. *P < 0.05 and **P < 0.01 were considered as statistically significant compared with agonist control.

ADP is a well-known soluble agonist for platelet aggregation and thrombus formation [2]. Previously, we showed that ADP at 10 *μ*M induced complete platelet aggregation and, therefore, this concentration was used to induce platelet aggregation in this study. PCME (5–20 *μ*g/mL) showed a dose dependent inhibition of ADP (10 *μ*M) induced platelet aggregation in rat platelets with a potent effect observed at the highest dosage (20 *μ*g/mL) used (Figure 2).

### 3.2. PCME Attenuated ADP-Induced [Ca^2+^]*i* Elevation and ATP Release

**Figure 3:**
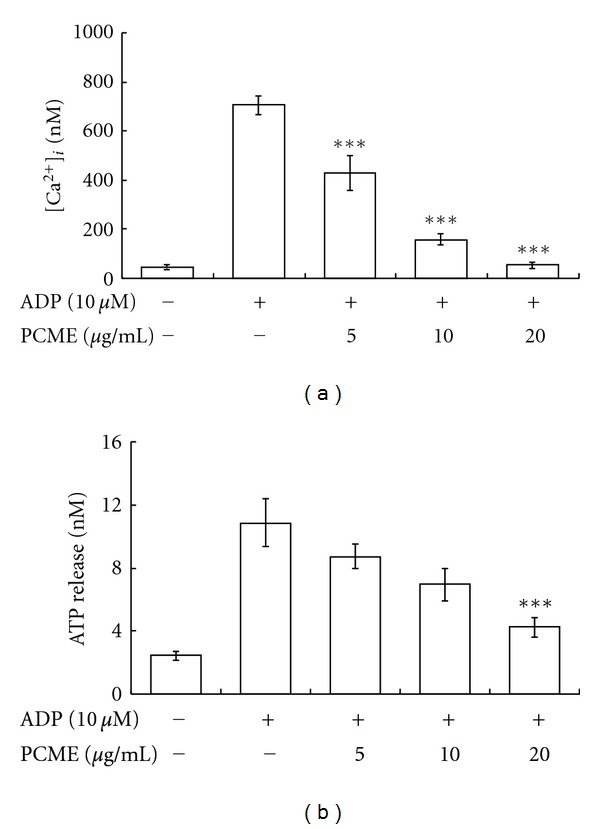
Inhibitory effect of PCME on ADP-induced [Ca^2+^]*i* mobilization and ATP release. Washed platelets were incubated with calcium fluorophore (fura-2/AM) and then stimulated with ADP as described in Section 2 for [Ca^2+^]*i *determination (a). For ATP assay (b), washed platelets were pre-incubated with PCME, stirred in an aggregometer for 3 min and then stimulated with ADP for 5 min. Each graph shows mean ± SEM from 3 independent experiments performed. ***P < 0.01 was considered as statistically significant compared with agonist control.

Platelet activation is marked by the release of platelet granular contents. Therefore, we determined whether PCME used at various concentrations attenuated the release of [Ca^2+^]*i* and ATP from dense granules in platelets stimulated with ADP. PCME (5–20 *μ*g/mL) strongly diminished [Ca^2+^]*i* mobilization (Figure 3(a)), and the same inhibitory effect on ATP release was observed at a lesser degree (Figure 3(b)). 

### 3.3. The Effect of PCME on TXA2 and cAMP Generation in ADP-Activated Platelets

**Figure 4:**
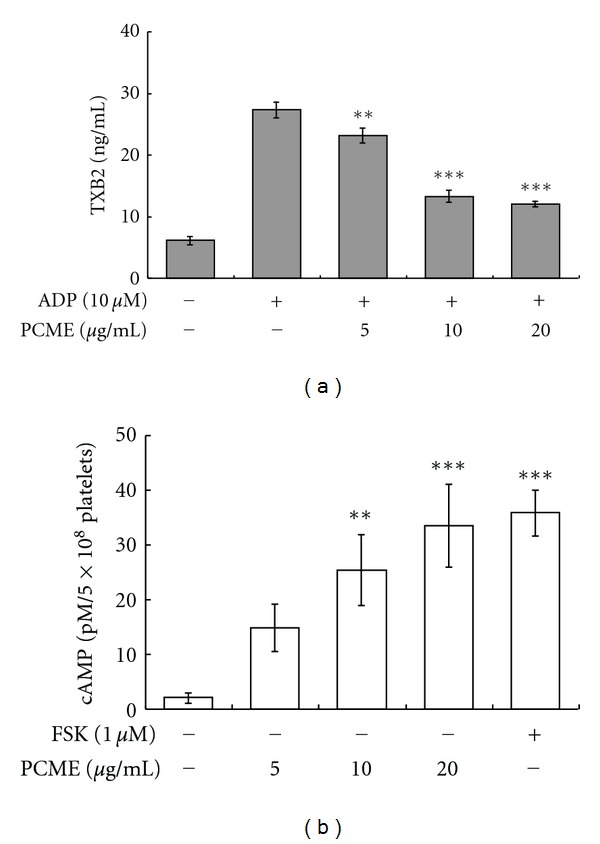
Effect of PCME on TXA2 generation and cAMP production. ADP (10 *μ*M) evoked TXB2 production (as measure of TXA2 generation) which was measured after 5 min of ADP stimulation according to EIA Kit instructions (a). For cAMP assay (b), washed platelets were stirred with vehicle, PCME, or forskolin in an aggregometer prior to ADP stimulation for 5 min. Reaction was terminated, and then cAMP enzyme immunoassay was performed as described in Section 2. Bar graphs show mean ± SEM of 3 independent experiments performed. **P < 0.01 and ***P < 0.005 considered as statistically significant compared with control.

While thromboxane A2 (TXA2) is an important mediator in the amplification of platelet activation, cAMP plays an imperative role in antiplatelet activity. Therefore, we measured the intracellular level of ADP-induced TXA2 generation and the level of cAMP in unstimulated platelets pretreated with PCME *in vitro*. PCME (5–20 *μ*g/mL) markedly inhibited the stable form of TXA2 (i.e., TXB2) in ADP-stimulated platelets (Figure 4(a)) whereas the level of cAMP was profoundly upregulated and its level at the highest concentration of PCME (20 *μ*g/mL) was comparable with that of forskolin used at 1 *μ*M (Figure 4(b)). 

### 3.4. PCME Attenuated JNK, ERK, and Akt Phosphorylations in ADP-Induced Platelets

**Figure 5:**
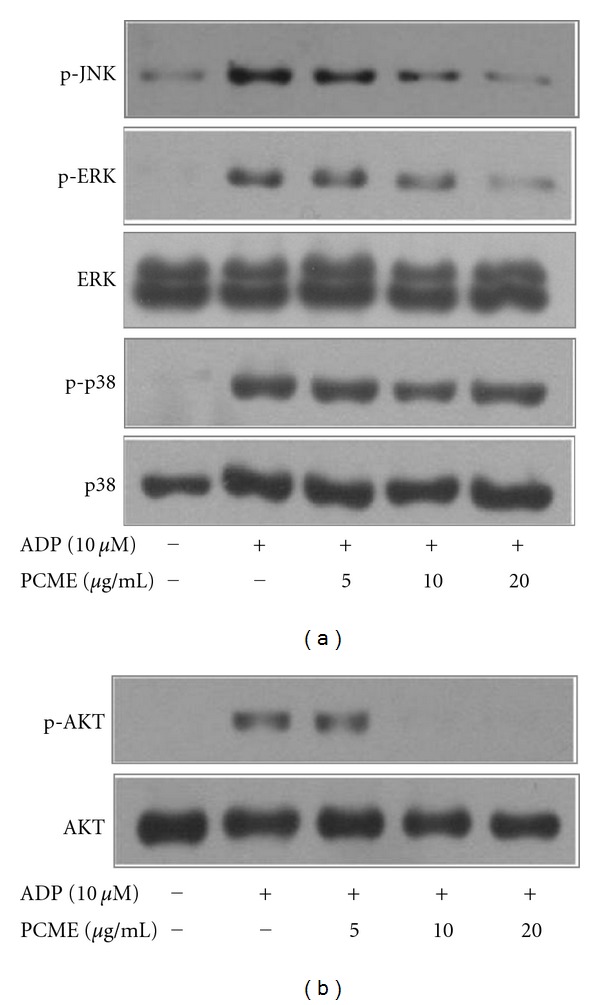
Effects of PCME on ADP-induced MAPK and Akt phosphorylations. Washed platelets were stirred in an aggregometer with vehicle or PCME for 3 min and then stimulated with ADP for 5 min. Reaction was terminated and proteins were extracted, separated by SDS-PAGE, blotted onto PVDF membrane and probed using antibodies against phospho- and total (JNK, ERK, p38) (a); and phospho- and total Akt (b). Bands represented triplicate of independent experiments.

Phosphorylations of mitogen-activated protein kinases (MAPKs) (ERK, p38, and JNK) and Akt in platelets are closely associated with platelet activation and aggregation. Hence, we determined if PCME inhibited MAPK and Akt phosphorylations in ADP stimulated platelets. Whereas p38 phosphorylation was unaffected by PCME (5–20 *μ*g/mL), ERK and JNK phosphorylations were dose-dependently suppressed in ADP-activated platelets (Figure 5(a)). Moreover, PCME totally demolished phosphorylation of Akt beyond 5 *μ*g/ml of concentration (Figure 5(b)). 

### 3.5. PCME Inhibited Fibrinogen Binding to Integrin *α*_IIb_ *β*_3_ in ADP-Activated Platelets

**Figure 6:**
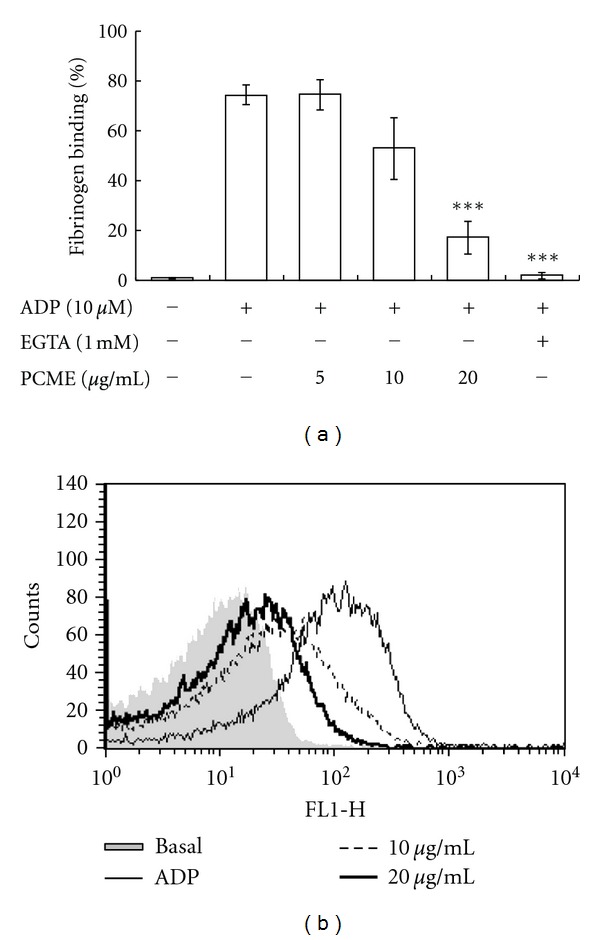
Effects of PCME on fibrinogen binding to integrin *α*_IIb_ *β*_3_ in ADP-activated platelets.Washed platelets were pretreated with PCME at 10 and 20 *μ*g/mL, and then stimulated with ADP (10 *μ*M) in Alexa Fluor 488-human fibrinogen (20 *μ*g/mL) for 5 min, fixed with 0.5% paraformaldehyde at 4°C for 30 min. Fluorescent intensity graphs indicate 4 independent experiments (a). Data exhibit mean fluorescence intensity (MFI) of fibrinogen-positive platelets. Each graph was expressed as % of gated (b). **P < 0.01 and ***P < 0.005 were considered as statistically significant compared with agonist.

Integrin activation and the subsequent fibrinogen binding to *α*_IIb_ *β*_3_ are crucial events in platelet aggregation. Thus, we determined whether fibrinogen binding to integrin *α*_IIb_ *β*_3_ was inhibited in platelets pretreated with PCME and stimulated by ADP. Our finding revealed that PCME at 10 and 20 *μ*g/mL inhibited fibrinogen binding to activated integrin *α*_IIb_ *β*_3_ in ADP-stimulated rat platelets (Figures 6(a) and 6(b)).

## 4. Discussion

In this study, we reported that *Pistacia chinensis* methanolic extract (PCME) displayed broad inhibitory effects on platelet aggregation, calcium mobilization, ATP release, TXA2 formation, and fibrinogen binding to *α*_IIb_ *β*_3_ in ADP-activated platelets. Moreover, PCME enhanced cAMP production in unstimulated platelets whereas it downregulated ADP-stimulated ERK, JNK, and Akt phosphorylations. 

ADP activates two G-protein-coupled purinoceptors, P2Y_12_ and P2Y_1_, to transduce its intracellular signaling [15]. P2Y_1_ receptor is linked to Gq to activate PLC*β* with consequent formation of inositol-1,4,5-trisphosphate (IP3) and Ca^2+^ release in one side and 1,2-diacylglycerol required for protein kinase C (PKC) activation on the other. P2Y_12_ receptor is linked to the adenylate cyclase inhibiting Gi-protein [16]. Here, therefore, we explored that PCME inhibited the rise of [Ca^2+^]*i* in ADP stimulated platelets (Figure 3(a)) while it upregulated the level of cAMP in resting platelets (Figure 4(b)), suggesting that ADP receptors could be among the potential targets of PCME via upregulation of cAMP which has been reviewed as inhibitor of G-protein-coupled receptors [17], albeit this needs further insightful verification. Likewise, previous reports indicated that resorcinol showed antiplatelet activity [18] mediated through COX-1 inhibition [19], implicating that the diminished release of TXA2 (Figure 4(a)) at this moment might be originated from a similar effect of resorcinol available in PCME. 

Phosphoinositide 3-kinase (PI3K) and Akt play platelet activation via sequential activation of PI-3K/Akt, nitric-oxide synthase 3, soluble guanylate cyclase (sGC), and cGMP-dependent protein kinase [20]. Thus, PCME-mediated inhibition of Akt (Figure 5(b)) may unravel the potential anti-platelet activity of this extract that could be mediated through modulation of PI-3K/Akt/cGMP signaling path-way despite that cGMP has also been reported to inhibit platelet activation [17]. Moreover, a recent report indicated that PI-3K*β* plays an important role in ADP-induced ERK activation and TXA2 generation [7], implying that PCME-mediated inhibition of ERK phosphorylation and TXA2 release might indicate its potential target at P2Y_12 _/PI-3K/Akt signaling. Likewise, Adam et al. reported that JNK1^−/−^ platelets showed impaired platelet secretion that brought altered integrin *α*_IIb_ *β*_3_ activation and reduced platelet aggregation via a mechanism involving PKC [21]. Therefore, we speculated that the reduced fibrinogen binding to *α*_IIb_ *β*_3_ (Figures 6(a) and 6(b)) could be partly attributed to JNK1 inactivation (Figure 5(a)) that might be linked to impaired P2Y_12_-dependent PKC function in PCME pretreated platelets. 

We conclude that PCME inhibited ADP-stimulated rat platelet aggregation, JNK, ERK, and Akt phosphorylations. While PCME downregulated TXA2 release and fibrinogen binding to *α*_IIb_ *β*_3_ in activated platelets, it showed enhanced cAMP production at the resting state. Thus, PCME could be considered as a potential antiplatelet agent targeting ADP amplified second wave of platelet secretion and aggregation. 

## Figures and Tables

**Table 1:** List of chemicals identified in PCME using GC-mass spectrometry.

Name of compound	(%)
Methane	66.4
DMSO	11.4
Dichloroethylene	1.1
Resorcinol	18.9
Pyridine	2.2

## Abbreviations

PCME: Pistacia chinensis methanolic extract
MAPK: Mitogen-activated protein kinase
TXA2: Thromboxane A2
GPCR: G-protein-coupled receptor
PKA: Protein kinase A
ADP: Adenosine diphosphate
ERK: Extracelluar signal-regulated kinase
JNK: c-jun N-terminal kinase.
